# Spatial and Temporal Freezing Dynamics of Leaves Revealed by Time‐Lapse Imaging

**DOI:** 10.1111/pce.15118

**Published:** 2024-09-10

**Authors:** Cade N. Kane, Scott A. M. McAdam

**Affiliations:** ^1^ Department of Botany and Plant Pathology Purdue University West Lafayette Indiana USA; ^2^ Department of Organismic and Evolutionary Biology Harvard University Cambridge Massachusetts USA

**Keywords:** frost, frost tolerance, honeysuckle, leaf, lonicera, xylem

## Abstract

Freezing air temperatures kill most leaves, yet the leaves of some species can survive these events. Tracking the temporal and spatial dynamics of freezing remains an impediment to characterizing frost tolerance. Here we deploye time‐lapse imaging and image subtraction analysis, coupled with fine wire thermocouples, to discern the in situ spatial dynamics of freezing and thawing. Our method of analysis of pixel brightness reveals that ice formation in leaves exposed to natural frosts initiates in mesophyll before spreading to veins, and that while ex situ xylem sap freezes near 0°C, in situ xylem sap has a freezing point of −2°C in our model freezing‐resistant species of *Lonicera*. Photosynthetic rates in leaves that have been exposed to a rapid freeze or thaw do not recover, but leaves exposed to a slow, natural freezing and thawing to −10°C do recover. Using this method, we are able to quantify the spatial formation and timing of freezing events in leaves, and suggest that in situ and ex situ freezing points for xylem sap can differ by more than 4°C depending on the rate of temperature decline.

## Summary

Freezing dynamics in leaves of *Lonicera × purpusii* captured with time‐lapse imaging in situ.

## 1 Introduction

Freezing air temperatures mark the end of woody‐plant primary productivity at high latitudes (Baldocchi et al. 2005). To survive this seasonal extreme, deciduous species shed senesced leaves and enter a leafless dormant state (Bassow and Bazzaz 1998; Clements and Ludlow 1977; Lubbe and Henry 2019; Sakai and Larcher 1987), while the leaves of evergreen and brevideciduous species tolerate repeated freezing and thawing cycles through all, or most of, the winter, respectively (Koehler, Center, and Cavender‐Bares 2012; Taneda and Tateno 2005). The ability of plants to retain leaves that can survive multiple freeze‐thaw cycles ensures carbon assimilation during brief warm periods through winter and in early spring (Chabot and Hicks 1982; Hughes and Smith 2007; Miyazawa and Kikuzawa 2005; Sprugel 1989). Consequently, leaf freezing tolerance is adaptively relevant and can determine plant community composition at high latitudes (Inouye 2000; Löffler 2007; Stuart et al. 2007; Tranquillini 1982; Walker et al. 2004). As average temperatures trend warmer, a shift towards earlier bud burst in temperate deciduous forests may increase the exposure of sensitive species to spring frosts normally avoided due to longer bud dormancy (Lamichhane 2021; Menzel, Helm, and Zang 2015). The economic consequences in horticulture of these changes are considerable, where leaves and flowers are at a risk from sudden frosts (Rodrigo 2000; Zohner et al. 2020). Despite the risks of freezing to plants, studies investigating the mechanism of freezing survival in situ have declined significantly in the last 20 years (Kaya et al. 2021; Wisniewski et al. 2009), such that we still do not have a good understanding of the spatial pattern of freezing in leaves, or whether the rate of freezing determines the ability of leaves to recover photosynthetic function on thawing (Ishikawa et al. 2016; Kaya et al. 2021; Rodrigo 2000; Stegner, Wagner, and Neuner 2020).

Leaves that can survive multiple freezing events do so by one of two major strategies, by supercooling organs to avoid ice nucleation (Sakai and Larcher 1987), or tolerance of multiple freeze events (Burke et al. 1976). The supercooling response, or frost avoidance strategy, provides transient protection for plants (Ball et al. 2002; Larcher et al. 1991; Rada et al. 1987; Sakai and Larcher 1987; Squeo et al. 1991; Thomas and Barber 1974). This strategy is widely adopted by plants that experience infrequent freezing events, but fails in freezing protection if the plant experiences temperatures beyond the supercooling threshold limit (Arias et al. 2015; Sakai and Larcher 1987; Squeo et al. 1991). The other strategy employed by plants to survive freezing temperatures is tissue being able to tolerate or recover from multiple freezing events (Cochard et al. 2001; Davis, Sperry, and Hacke 1999; Feild and Brodribb 2001; Sakai and Larcher 1987; Sperry and Sullivan 1992; Sucoff 1969). This involves cell survival from apoplast localized freezing sometimes referred to as ice accommodation (Ishikawa et al. 2022; Stegner, Lackner, et al. 2020; Stegner, Wagner, and Neuner 2020). When leaf freezing occurs, apoplast freezing generates a brief temperature spike referred to as the first exotherm, followed by a second exotherm when the cell sap freezes, once the second exotherm occurs it is assumed cell function is irrecoverable (Ashworth 1993; Sakai and Larcher 1987; Squeo et al. 1991; Stergios and Howell 1973; Taschler and Neuner 2004). It is well known that some plants will osmotically adjust cells during the autumn and winter to reduce the temperature threshold of symplastic freezing (Gail 1926; Levitt 1957; O'Neill 1983; Pramsohler and Neuner 2013). The primary causes of freezing damage to cells in woody plants is by ice nucleation piercing cell membranes (Guy 1990), extracellular ice formation causing cell dehydration (Gusta, Burke, and Kapoor 1975) or freezing and thawing in the xylem tissue inducing embolism that breaks the water transport stream, leading to dehydration and death of downstream tissues (Koehler, Center, and Cavender‐Bares 2012; Langan, Ewers, and Davis 1997; Mayr, Gruber, and Bauer 2003; Sperry et al. 1994; Utsumi et al. 1999). Rapid freezing is also known to induce freezing‐damage (Burke et al. 1976; Weiser 1970), but much less is known about the impact of thawing speed on tissue survival (Arora 2018).

The most common method to measure plant freezing temperatures, the timing of freezing and freezing progression across organs is infrared video thermography where temperature is measured using the output of thermal energy in the form of infrared radiation (Fuller and Wisniewski 1998; Stier et al. 2003). This technique allows for relatively easy analysis of plant tissue freezing in many types of plant tissue, but does require relatively costly, specialized cameras (Fuller and Wisniewski 1998; Morales, Sierra‐Almeida, and Kalin Arroyo 2023). Another way that in situ freezing has been monitored in the field is via nuclear magnetic resonance microscopy which uses magnetic resonance to observe state changes in, predominantly, excised plant tissues as liquid water becomes ice (Hills and Remigereau 1997; Ide et al. 1998). This technique has a very high spatial, tissue‐scale of resolution, but is expensive and challenging to deploy in the field (Hills and Remigereau 1997; Ide et al. 1998; Ishikawa et al. 2009).

In this study, we utilized the brevideciduous Purpus honeysuckle (*Lonicera × purpusii* Rehder. [Caprifoliaceae]) which is a sterile F1 hybrid between *L. fragrantissima* Lindl. & Paxton and *L. standishii* Jacques (Dulić 2012; USDA 2022) to examine the freezing point of leaf tissues and ex situ xylem sap. The genus *Lonicera* contains multiple species of shrub, liana and creeper that are highly invasive, particularly in eastern North America (Love and Anderson 2009; Miller and Gorchov 2004; Schierenbeck 2004), as well as freezing tolerant (Brailko and Gubanova 2014; McEwan et al. 2009; Tofig, Shalala, and Aisel 2022). One of the primary causes of Eastern Asian *Lonicera* spp. Naturalization in Eastern North America is the ability of leaves of these species to tolerate mild freezing conditions in both spring and autumn which allows for a prolonged growing season when compared to native shrubs, increasing annual assimilation while overstory trees are leafless (Fridley 2012; McEwan et al. 2009; Smith 2013). To investigate the nature of freezing in *L. × purpusii* we adapted a time‐lapse imaging apparatus originally developed to quantify embolism spread in xylem, using a RaspberryPi driven camera and manifold to observe the progression and timing of natural winter freezing and thawing events in the field in leaves of *L. × purpusii* (Brodribb, Bienaimé, and Marmottant 2016; Brodribb, Skelton, et al. 2016). Our method based on analyzing pixel brightness to map the spatial patterns of freezing and thawing in leaf tissue can be used in the field to track freezing in tissues exposed to natural winter frost events. In addition to mapping freezing and thawing events in leaves, we also tested the effect of rate of temperature decline and rate of thawing on leaf photosynthetic recovery and damage in this species.

## 2 Materials and Methods

### 2.1 Plant Material

A 10‐year‐old specimen of *L. × purpusii* was used for all experiments, grown outside on the campus grounds of Purdue University, West Lafayette, IN, 47907, USA (40.422833 N, −86.916837 W) on the South facing side of a building. Measurements were taken between January and March 2021. For experiments conducted in the lab on excised branches, stems longer than the longest vessel were always used to avoid inducing embolism. The mean length of the longest vessel, determined by air‐injection, was 34.5 ± 1.96 mm (*n* = 4).

### 2.2 Visualizing Leaf Freezing

To visualize the freezing of leaves Raspberry Pi 4 Model B (Raspberry Pi, United Kingdom) clamps were used (Brodribb, Skelton, et al. 2016). To capture freezing events in situ, the Raspberry Pi clamp was attached to an unfrozen, green leaf still attached to the shrub on the afternoon of days when temperatures were above freezing, but before nights when minimum temperatures were forecast to drop below −20°C, and also forecast to rise above freezing the following day (Day of the Year [DOY] 27, 28 and 33) (Supporting Information S1: Figure S2A). Representative data is shown from the leaf measured over the night of DOY 28. Leaves were imaged every 3 min. Leaf temperature was monitored using a fine wire thermocouple placed on the leaf surface inside the clamp and attached to the CR850 data logger (Campbell Scientific, Utah, USA). To analyse freezing dynamics, images were assembled into an image stack using ImageJ image analysis software (US National Institutes of Health, Maryland, USA).

The image stack was divided into a 448 × 448‐pixels section which included midrib, minor veins and mesophyll in a 25 mm^2^ field of view (FOV). This stack was then divided into 196 further divisions (32 × 32‐pixels, or a FOV of 0.125 mm^2^) and mean pixel brightness (mean RGB value for the whole section) was extracted from each slice of all 196 subsections. The onset of freezing was determined as an increase in mean pixel brightness of 10% of the difference between initial and maximum mean pixel brightness, and onset of thawing was determined by a decrease from maximum pixel brightness of 10% of the difference of initial and maximum mean pixel brightness (Supporting Information S1: Figure S2C). To test the spatial limits of this method a 90 × 90‐pixel section (FOV 1 mm^2^) was analysed in the same way with each subsection being 9 × 9‐pixels (FOV 0.01 mm^2^) focusing on an areole (Supporting Information S1: Figure S2B). The raw leaf temperature data collected with a fine wire thermocouple attached to the leaf during freezing and thawing was fitted with a linear regression to determine the temperature at which each subsection froze or thawed based on changes in image/pixel brightness. In all ex situ experiments, both fine wire thermocouples and imaging were used, with freezing points determined from both exotherms and by analyzing the average pixel brightness for the FOV of each stack 1920 × 1080‐pixels. Additionally, we analysed all collected image stacks for possible freeze‐thaw embolism according to the optical vulnerability image subtracted method from Brodribb, Skelton, et al. (2016) and opensourceov.org.

### 2.3 Determination of the Freezing Point of Leaf, Stem and Xylem Sap

To determine freezing points, fine‐wire thermocouples attached to leaves (*n* = 3) and connected to a CR850 data logger were used to measure temperature, logged every 1 s. Thermocouples were folded so they remained in constant contact with the leaf surface. To measure intact xylem freezing points (*n* = 3), bark and cambium were carefully removed by hand to avoid causing embolism in the underlying xylem and the exposed area was washed with deionized water to eliminate cellular contents from the phloem and cambium. Thermocouple wires were then tied around the stems with the tip of the thermocouple placed against the exposed xylem tissue of a branch internode. Ex‐situ xylem sap was collected from branches (*n* = 3) with at least 10 leaves. The cambium and phloem tissues were removed from the cut end to reveal the xylem, and the stems were then placed into a Scholander Pressure Chamber (PMS Instrument Company, Oregon, USA), which was laid on its side and gently pressurized using N_2_ gas until 0.05 MPa beyond the endpoint when xylem sap began to flow from the cut end (approximately 0.3 MPa of pressure). The sap, approximately 1 mL, was collected in 2 mL tubes over 10 min, after initially wiping the cut end to remove any potential contaminants from excision.

To determine freezing points, all tissues were exposed to the same treatment. Samples were placed into a large plastic zip‐lock bag that contained a damp paper towel to reduce evaporation. This bag was then placed into a Styrofoam box to provide a uniform air temperature and slow temperature declines, the box was then placed into a −10°C freezer (Roper Technologies, Florida, USA). The time to freezing took approximately 60 min, with an average cooling speed of −1.6°C ± 0.3 min^−1^. Temperatures were recorded until after the freezing exotherm. After observing the exotherm, the samples were left in the freezer to ensure no additional freezing events occurred. The freezing point was determined to be the highest temperature recorded immediately after the freezing exotherm (Beck et al. 1984; Woo and Mujumdar 2010). Three samples of each tissue type or xylem sap were used to determine the mean freezing point.

### 2.4 The Effect of Cooling and Warming Rate on Recovery

To test the effect of how the rate of temperature decline and increase impacts photosynthetic recovery in *L. × purpusii*, branches longer than the longest vessel were excised under deionized water and leaves placed in plastic bags containing damp paper towels. Two high power Schott KL 2500 LCD lights (Schott AG, Germany) were used to irradiate the branches for 1 h (providing a light intensity of 500 PAR at the leaf surface). After the hour of high light exposure gas exchange in three leaves from two branches was measured using a LI‐6800 Portable Photosynthesis System (LI‐COR Biosciences, NE, USA) set to a vapour pressure deficit of 1.2 kPa, CO_2_ of 400 ppm and light intensity of 1500 PAR. Fine wire thermocouples were then attached to leaves with tape so temperatures could be monitored via the CR850 data logger. Branches were then re‐bagged with a damp paper towel and either placed directly into a −10°C freezer in which leaves were frozen rapidly (−11.4°C ± 0.82 min^−1^) or placed inside a Styrofoam box and then placed into a −10°C freezer, to freeze slowly (−1.6°C ± 0.3 min^−1^). Leaves were then allowed to warm to room temperature in the air ( + 11.9°C ± 1.02) or in a Styrofoam box ( + 1.4°C ± 0.08). Leaf temperatures were monitored as they declined and the freezing exotherm was noted. We allowed temperature to fall after the exotherm to around −10°C. Branches were then removed from the freezer and allowed to warm either rapidly in the open air ( + 11.9°C ± 1.02 min^−1^) or in the Styrofoam box ( + 1.4°C ± 0.08 min^−1^), recut under water, after which they were and put back under high light for 1 h and photosynthesis was remeasured in the same leaves using the LI‐6800 using the same environmental settings. Cooling and warming rates for measured leaves were determined by finding the slope of a linear regression plotted through the leaf temperature after it was placed in or removed from the freezer. For leaves measured in situ, this rate was determined by finding the slope of a linear regression plotted though the observed temperatures between 6 and 8 pm DOY 28, 2021.

Leaf water potential was measured in neighbouring leaves (*n* = 6) on the same branch immediately after measuring photosynthesis before and after thawing by wrapping leaves in a damp paper towel and placing them in a sealed plastic bag and allowing to equilibrate in a dark drawer for 10 min before measurements were made in a Scholander Pressure Chamber (PMS Instrument Company, Oregon, USA).

### 2.5 Assessing Maximum Photosynthetic Rates of Leaves Exposed to Air and Buried Under Snow Under Repeated Freezing and Thawing

To test whether damage occurred to leaves by freezing or senescence, and the lowest temperature from which *L. × purpusii* leaves could survive after freezing, maximum photosynthetic rates were measured in three leaves on 16 days over the 26 days of observation starting on DOY 28, 2021 which preceded a winter snowstorm on DOY 30, 2021 (during which 150 mm of snow fell over 48 h), and until snow melted on DOY 54, 2021. After the snowfall, lower branches with leaves buried by the snow were marked with tape for measurement of leaves upon snow melt. To measure maximum assimilation rate, branches longer than the longest vessel were cut under deionized water, bagged with a zip top bag containing damp paper towels and transported to the lab. Once inside the branches were placed under lights for 1 h then gas exchange in three leaves was measured in a LI‐6800 set to a vapour pressure deficit of 1.2 kPa, CO_2_ of 400 ppm and light intensity of 1500 PAR and temperature of 22°C. All branches measured before the snowstorm were exposed to the air for the duration of the measuring window. After the snowstorm, the upper branches that were exposed to the air were compared to leaves on lower limbs that were buried under snow since the snowfall event. Minimum nighttime air temperatures were recorded at the Purdue University Airport located 2 km from the study plant. Long term instantaneous leaf gas exchange data from this individual of *L. × purpusii* for the same growing season (2020−2021) is published in Kane and McAdam (2023).

### 2.6 Data Analysis and Statistics

The freezing temperatures of leaf, xylem and ex‐situ xylem sap and the recovery of leaf assimilation and water potential were analysed using a one‐way ANOVA with a Tukey's HSD post hoc test assuming normal distribution, from multiple measurements of the same individual. The freezing temperature of veins and mesophyll in the 90 × 90 section were compared using two‐way *t*‐tests by averaging the freezing initiation temperature calculated from changing brightness for sections of leaf that contained vein or only mesophyll. Percent changes in water potential and assimilation was calculated by subtracting the initial from final leaf water potential and assimilation before and after freezing at different rates of temperature decline and temperature increase, then dividing the difference by the initial value and multiplying by 100. Significance was determined using a one‐way ANOVA with a Tukey's HSD post hoc test.

## 3 Results

### 3.1 High Resolution Evaluation of Leaf Freezing Using Time‐Lapse Imaging

**Figure 1:**
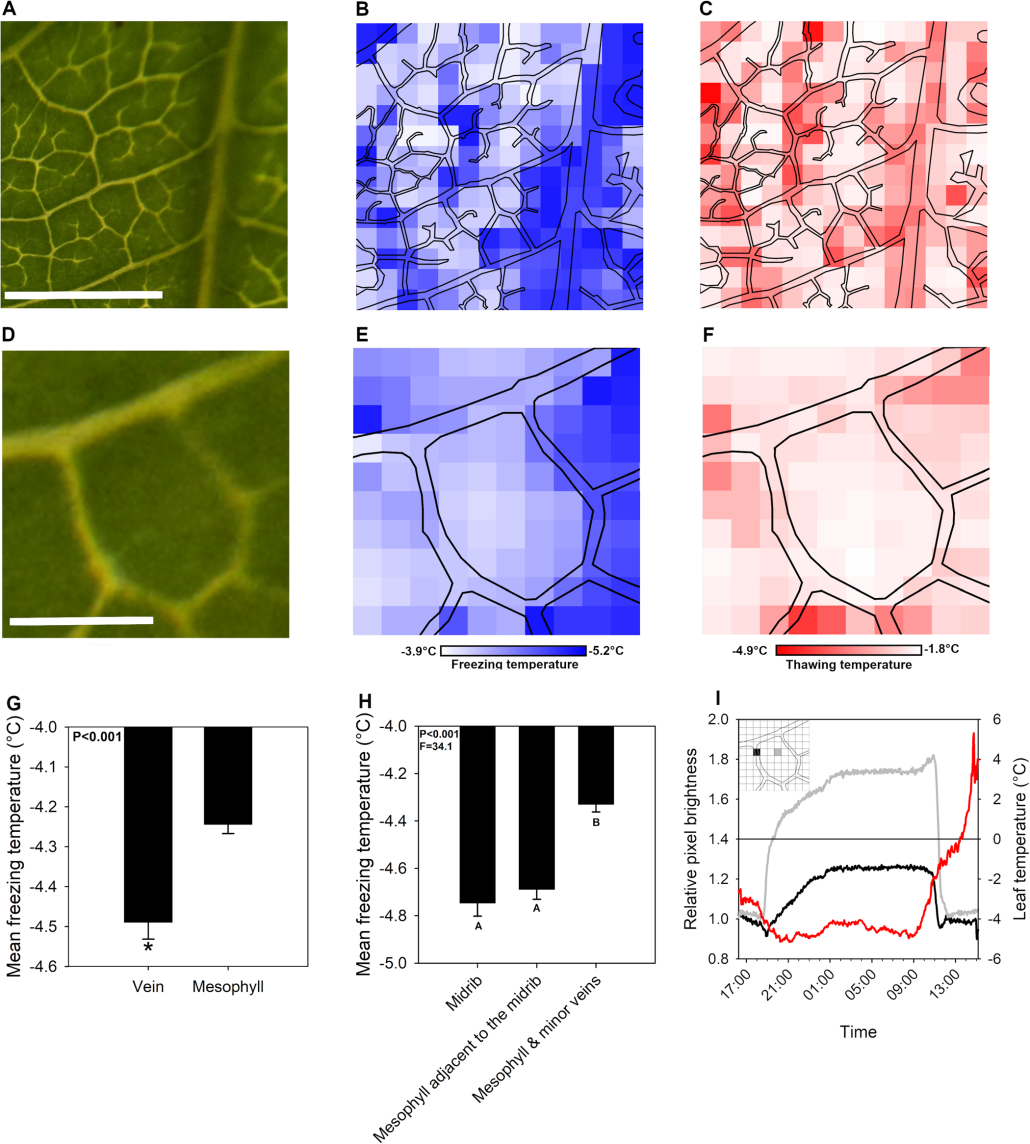
(A) An image of the unfrozen area of *Lonicera × purpusii* leaf that was tracked through an in situ overnight, freeze‐thaw cycle on DOY 28, 2021 (scale bar = 2.5 mm). (B) The spatial distribution of freezing temperatures and (C) subsequent natural thawing temperatures of individual 0.125 mm^2^ regions of the same field of view. The temperature at which brightness increased or decreased by 10% was used to determine freezing and thawing temperatures for each section of the leaf. Veins are outlined in black in (B) and (C). (D) An image of an unfrozen areole of the same leaf which was analysed at a finer spatial scale through an in situ overnight, freeze‐thaw cycle (scale bar = 0.5 mm). (E) The spatial distribution of freezing temperatures and (F) subsequent natural thawing temperatures for 0.01 mm^2^ regions of the same areole. Colour scales indicate freezing (B and E) and thawing (C and F) temperatures, respectively. (G) The mean freezing temperature of 0.01 mm^2^ sections of the leaf that contain veins or only mesophyll, ‘*’ indicates a significant difference based on a two‐way Student *t‐*test. (H) The mean freezing temperature of 0.25 mm^2^ sections of leaf that comprise the midrib, mesophyll adjacent to the midrib and mesophyll and minor veins distant from the midrib, letters denotes significant differences in means based on a one‐way ANOVA and a Tukey's HSD post hoc test (*p* < 0.05). (I) The nocturnal course of leaf temperature (red) and relative pixel brightness of a 0.01 mm^2^ section of the leaf containing mostly vein (black) or mesophyll (grey). The horizontal line marks 0°C. The insert depicts the area of the leaf from (E) in which the brightness was measured with the black pixel being the section of the leaf containing the vein and the grey section of the leaf from the mesophyll.

By converting pixels to brightness through the time‐lapse image stack taken of a leaf exposed to a natural freezing and thawing during a winter night, we were able to identify a clear transition between unfrozen, frozen and then subsequently thawed leaf tissue, including in veins and mesophyll (Figure 1A; Video S1). Analysis of the time‐lapse series of images identified a vivid spatial pattern of freezing during the night, with the first pixels initiating freezing at −3.95°C while the final pixels initiating freezing at −5.07°C (Figure 1B). Freezing was initiated in the mesophyll at higher temperatures than the midrib, with midrib freezing occurring at −4.74°C ± 0.06, while freezing in the mesophyll and minor veins not adjacent to the midrib occurring at −4.33°C ± 0.03 (Figure 1B). Mesophyll closest to the midrib froze at lower temperatures similar to those of the midrib, at −4.69°C ± 0.04 (one‐way ANOVA, *p* < 0.001, *F* = 34.1) (Figure 1B). When a small 1 mm^2^ area of leaf spanning an areole, not adjacent to the midrib, was divided into 100 pixels and analysed, the freezing of pixels in the mesophyll not intercepted by veins occurred at −4.24°C ± 0.02, while pixels intercepted by veins froze at a lower temperature of −4.49°C ± 0.04 (two‐way *t*‐test, *p* < 0.001) (Figure 1E). Thawing occurred at less negative temperatures than freezing occurred, between −4.81°C and −1.89°C (Figure 1C). We observed similar spatial relationships between tissue types during thawing with the midrib thawing earlier than the mesophyll and minor veins (Figure 1C and Supporting Information S1: Figure S1B) in the 25 mm^2^ FOV. We also observed in the 1 mm^2^ FOV areole section that thawing was initiated in the veins and occurred at a higher temperature in the mesophyll (Figure 1F and Supporting Information S1: Figure S1C).

### 3.2 Effect of Freezing and Thawing Rate on Photosynthetic and Water Potential Recover

**Figure 2:**
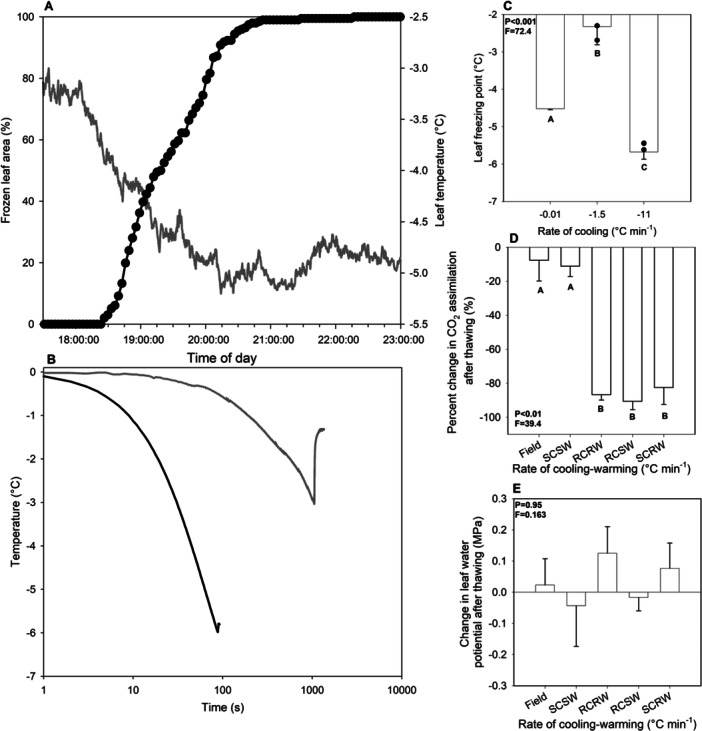
(A) The percentage of leaf area imaged that froze (black) over the course of the night on DOY 28, 2021 and leaf temperature on the same night (grey). (B) Freezing exotherms of ex situ leaves frozen at −1.5°C min^−1^ (grey) and −11°C min^−1^ (black) versus the time in seconds after the leaves reached 0°C. (C) Freezing temperatures determined by exotherms (bars) or pixel brightness (black points) of a leaf frozen at −0.01°C min^−1^, −1.5°C min^−1^, and −11°C min^−1^ with standard errors. Letters denotes a significantly different mean based on a one‐way ANOVA and a Tukey's HSD post hoc test (*p* < 0.05). (D) Mean ( ± SE) of photosynthetic recovery of leaves cooled and warmed in the field (at −0.01°C min^−1^ and +0.02°C min^−1^ [*n* = 3]) or ex situ cooled and warmed slowly (slow cooling‐slow warming [SCSW] at −1.5°C min^−1^ and +1.5°C min^−1^ [*n* = 6]), or cooled and warmed rapidly (rapid cooling‐rapid warming [RCRW] at −11°C min^−1^ and +11°C min^−1^ [*n* = 6] or either rapidly cooled and slowly warmed [RCSW] or slowly cooled and rapidly warmed [SCRW] [*n* = 3 each]). Letters denotes a significantly different mean based on a one‐way ANOVA and a Tukey's HSD post hoc test (*p* < 0.05). (E) Mean ( ± SE) change in leaf water potential of leaves cooled and warmed in the field or ex situ at different rates, described above. Letters denotes a significantly different mean based on a one‐way ANOVA and a Tukey's HSD post hoc test (*p* < 0.05).

Rate of temperature decline had a significant effect (one‐way ANOVA, *p* < 0.001, *F* = 39.4) on observed freezing temperature. In ex situ experiments conducted on leaves and branches in the lab, leaves cooled at −1.6°C min^−1^ and −11.3°C min^−1^ froze at a mean ( ± SE) temperature of −2.39°C ± 0.28 and −5.58°C ± 0.09, respectively (Figure 2). There was no difference between the freezing points determined by exotherms or average pixel brightness in these experiments (Figure 2C). When leaves were observed in situ in the field, the freezing point was found to be −4.52°C ± 0.024 according to pixel brightness analysis (Figure 2C). Freezing exotherms in leaves frozen at −1.4°C min^−1^ and −11.3°C min^−1^ happen 1048 s and 86 s after leaf temperatures dropped to 0°C with the release of latent heat of freezing taking 255 and 5 s, respectively (Figure 2B). Leaves frozen in situ took approximately 4.4 h to freeze completely (Figure 2A).

**Figure 3:**
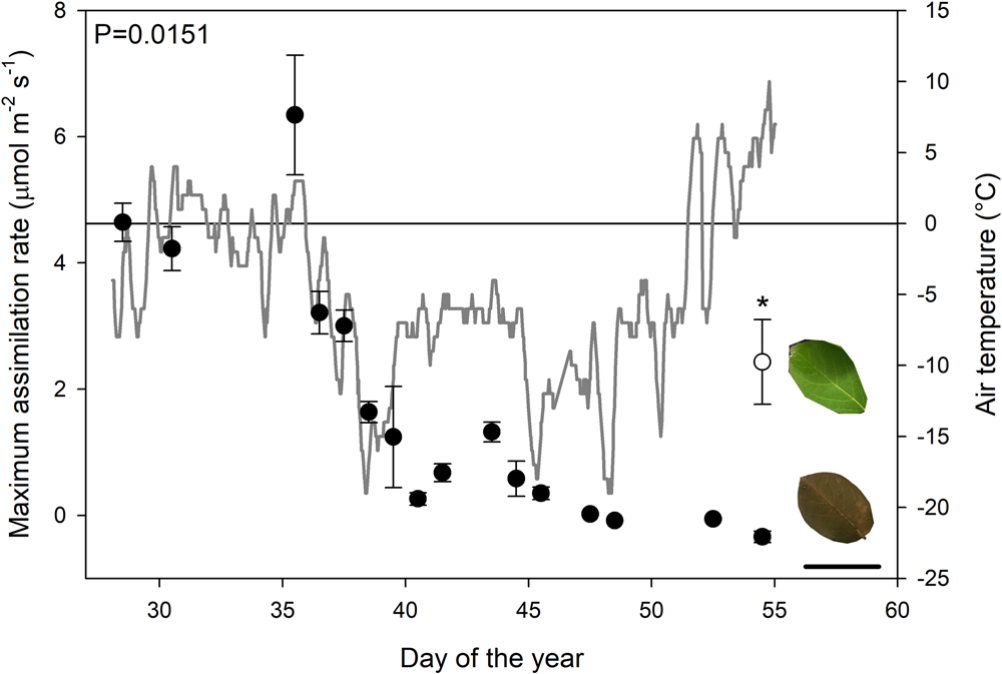
The mean maximum assimilation rate of leaves measured at 22°C that had been exposed to air during a snowstorm and cold period beginning DOY 30 (black points; *n* = 3), the white point represents the mean maximum assimilation rate measured on leaves from a branch that was buried under snow from the onset of the snowfall until the snow melted on the day of measuring (*n* = 3, ± SE). The grey line depicts air temperature. The ‘*’ denotes a significant difference between the maximum assimilation rates of the leaves exposed to air and buried under snow DOY 54. The black scale bar denoting 5 cm.

In ex situ experiments, we found that leaves cooled at −1.6°C min^−1^ and warmed at +1.4°C min^−1^ could survive freezing to −10°C, with only a minimal reduction in maximum assimilation rate measured on thawing (reduced by 11.1%) (Figure 2D). In contrast, rapid freezing and rapid thawing was found to permanently damage photosynthetic recovery in leaves exposed to temperatures that do not, or only minimally, damage photosynthetic capacity if frozen slowly (one‐way ANOVA, *p* < 0.001, *F* = 39.4) (Figure 3). Gas exchange of leaves that were cooled at −11.3°C min^−1^ or warmed at +11.9°C min^−1^ were severely compromised, with more than an 80% reduction in recovered assimilation rate (Figure 2D). We found that leaves cooled in situ at −0.01°C min^−1^ and warmed at 0.018°C min^−1^ (*n* = 3) had a similar recovery in photosynthetic rate to those frozen at −1.6°C min^−1^ with reductions in assimilation also of 7.7% (Figure 2D). Leaf water potential was not significantly affected by the rate of freezing (one‐way ANOVA, *p* = 0.95, *F* = 0.163) (Figure 2E). Ex situ freezing at −1°C min^−1^ resulted in similar freezing temperatures of leaf (−2.32°C ± 0.49) and exposed stem xylem (−2.14°C ± 0.5) while xylem sap froze at a significantly higher temperature (−0.36°C ± 0.084, one‐way ANOVA, *p* = 0.026, *F* = 7.1) (Supporting Information S1: Figure S1A).

### 3.3 Insulation by Snow Can Protect Leaves From Severe Freezing Events

Leaves in the field that had been exposed to minimum temperatures of −19°C in the air did not recover photosynthesis when thawed, compared to leaves of the same plant buried under snow (Figure 3). Warmed leaves that had been exposed to cold air respired, while leaves buried under snow were capable of positive assimilation rates with a mean of 2.43 ± 0.67 µmol m^−2^ s^−1^ (*n* = 3) *p* = 0.0151 (two‐tail *t*‐test) measured at midday. Low leaf water potentials were not the cause of photosynthetic failure in leaves exposed to the air, which had an average leaf water potential of −0.80 ± 0.64 MPa (*n* = 3), similar to the water potential of leaves buried under snow −0.57 ± 0.28 MPa (*n* = 3) *p* = 0.5994 (two‐way *t*‐test).

## 4 Discussion

### 4.1 Time‐Lapse Photography Can Capture the Spatial Dynamics of Freezing In Situ

Here we demonstrate a low cost, easily accessible method for capturing the pattern and timing of freezing and thawing in leaves by quantifying and analyzing changes in pixel brightness using timelapse imaging (Brodribb, Bienaimé, and Marmottant 2016), and fine‐wire thermocouples (Burke et al. 1976). This is similar to the earlier work of Kaku (1971), who, at a much higher spatial scale was able to determine the initiation point of whole leaf freezing. Image brightness has been used to track the growth and movement of ocean pancake ice floes (Roach, Smith, and Dean 2018) and can differentiate unfrozen and frozen leaves of alpine herbs (Solanki et al. 2022). Using this method, we observed that the midrib and the mesophyll that surround it freeze at the lowest temperatures in *L. × purpusii*, with lamina mesophyll and minor veins being the first tissues to freeze. A similar spatial pattern of initial mesophyll freezing, most distant from the major veins, has been observed in *Senecio incanus* and some leaves of *Buxus microphylla* (Hacker, Spindelböck, and Neuner 2008; Hacker and Neuner 2008; Kaku 1971). The process by which ice forms and spreads in leaf tissue appears to be species‐dependent, and can begin in the midrib in some species, particularly conifers (Hacker and Neuner 2008). In *Pinus mugo*, needles freeze in the midrib and the endodermis acts to keep ice from spreading to the mesophyll (Stegner et al. 2023). Other determinants of the tissue of initial freezing include leaf age, size and water content (Kaku 1971). Leaves that supercool during subzero air temperatures contain liquid water below 0°C. Only once ice nucleation begins at a nucleation site does ice spread through the leaf. The number of nucleation sites varies depending on leaf anatomy, microbial colonization and other unknown factors (Hacker and Neuner 2008; Kaku 1975; Wisniewski, Lindow, and Ashworth 1997). It has also been observed that plant water content can impact ice nucleation temperature with drier branches showing lower ice nucleation temperatures (Lintunen et al. 2018). Our method provides a quick and highly affordable means of examining the spatial and temporal dynamics of tissue freezing and thawing.

In *L. × purpusii*, we observed that the veins froze at the lowest temperatures (Figure 1). To test whether xylem sap has innately lower freezing points than mesophyll tissue we extracted xylem sap and determined the freezing point (Supporting Information S1: Figure S1A). We found that when xylem sap is extracted and frozen, the freezing temperature is near 0°C which agrees with earlier work on the freezing point of extracted xylem sap (Zimmermann 1964). This can be explained by the generally low osmotic content of xylem sap (Bollard 1960; Zimmermann 1964). With very little of the radial and axial volume in xylem tissue occupied by living parenchyma (Spicer 2014), even in the low‐density wood of *Lonicera* species (Ogata 1988), most of the volume of water in situ in the stem is assumed to be in xylem cells. This difference in temperature between the freezing point of veins and xylem sap suggests that some unknown aspect of water contained in vessel elements depresses the freezing point. More work is needed to address the question of why water under mild tension in vessels might be more resistant to freezing than that same water extracted from the conduits or, in the case of intact leaves, more resistant to freezing than the water in and around the mesophyll cells. Some studies have found correlations between vessel size and minimum freezing temperatures (Cavender‐Bares et al. 2005), suggesting that the vascular anatomy of a given species may play an important role in determining the freezing temperatures of the xylem. It is thought that the water in narrower vessels is more capable of reabsorbing expelled gas (expelled on freezing) when thawing (Sperry and Sullivan 1992; Utsumi et al. 1999). It has also been suggested, that differences in freezing embolism susceptibility based on vessel size may be due to larger vessels freezing at less negative temperatures than smaller vessels, leading to larger vessels being embolized by freezing (Cavender‐Bares et al. 2005; Lo Gullo and Salleo 1993). Our method for determining freezing dynamics and timing based on changes in pixel brightness, could be used to confirm possible relationships between xylem anatomy and freezing temperatures at a higher spatial scale similar to methods that require access to cryo‐scanning electron microscopy (Utsumi et al. 1999) or X‐ray microcomputed tomography (Charra‐Vaskou et al. 2023).

There are many ways that freezing can be visualized in plants including using infrared differential thermal analysis, where infrared images or video are used to observe changes in temperature and release of heat from ice formation to identify freezing temperature and spatial movement of ice (Larcher et al. 1991; Ping et al. 2023). Infrared microscopy can be used to visualize freezing at very fine resolution but is limited to a lab setting, when deployed in the field thermal cameras are typically used to visualize whole plant freezing responses (Livingston et al. 2018; Neuner et al. 2019; Stegner, Schäfernolte, and Neuner 2019). Other methods for visualizing freezing typically require expensive equipment or cannot be deployed in the field (Arora 2018; Stegner, Wagner, and Neuner 2020). These methods include cryo‐scanning electron microscopy which can be used to observe where ice forms in plant tissues, as freezing at lower temperatures typically produces larger ice crystals that can be distinguished from the small crystals that form when tissue is flash‐frozen using liquid nitrogen (Endoh et al. 2009; Fujikawa and Endoh 2014). Magnetic resonance imaging can determine the phase of water in tissues none destructively (Ide et al. 1998; Ishikawa et al. 2022, 2016). Similarly X‐ray phase contrast is able to discern the different phases of water in intact plant tissues based on X‐ray absorbance (Kovaleski, Londo, and Finkelstein 2019). Our method of analyzing timelapse image sequences of tissue freezing has a key advantage in being low‐cost, field deployable and noninvasive; while also providing a high spatial resolution.

Our work shows that the rate of temperature decline and increase is critical for the survival of *L. × purpusii* tissue. The rate of cooling and warming can determine the extent of tissue damage sustained by plants during freezing (Burke et al. 1976; Weiser 1970). In this study, we found that both rapid freezing and thawing cause damage to photosynthesis. It is unknown whether photoprotection pathways may influence this photosynthetic damage on rapid freezing and thawing given the importance of these pathways for freezing tolerance and survival (Liu et al. 2022). The ability of leaves to recover gas exchange capacity on thawing, and also surviving long periods frozen under snow, suggests that the xylem of frost tolerant *L. × purpusii* did not experience freeze thaw embolism, which would have damaged the water transport stream (Cardoso, Batz, and McAdam 2020; Skelton et al. 2017). Furthermore, our time‐lapse analysis did not detect any evidence of embolism forming in leaf veins of leaves exposed to a natural freezing and thawing cycle in *L. × purpusii*. The rate of temperature decline appeared to have a major impact on the observed freezing temperature of leaves. With three different cooling rates yielding three significantly different freezing temperatures (Figure 2C). Rapid cooling can cause an erroneous determination of bulk tissue freezing temperature, either by snap freezing resulting in a underestimate (Pearson and Davison 1993) or the rapid cooling causing faster supercooling than in nature, leading to and overestimate of freezing temperature (Salt 1966). When designing experiments to determine frost tolerance, survival and tissue freezing temperature we would recommend a slower cooling rate and thawing rate.

*L. × purpusii* leaves can recover function through repeated mild freezing events in a season with photosynthesis recovering on thawing, even when exposed to very low air temperatures (Figure 3). Leaves of *L. × purpusii* in situ were able to survive minimum nighttime temperatures above −19°C with minimal damage to maximum photosynthetic capacity. Once leaves were frozen below −19°C, upon thawing, tissue would turn brown and photosynthesis was unrecoverable. This closely matches with publicly available horticultural information for this species which suggests the lowest safe temperature in which to grow *L. × purpusii* is −20°C (www.rhs.org.uk). Lower branches buried in snow during the coldest days of winter were protected, remaining green on thawing and could still undertake photosynthesis. This colour change in damaged leaves upon thawing may mean that extending time lapse analysis may offer insights into damage experienced during freeze thaw cycles. Snow is well recognized as an insulator against severe air freezing (Briceño et al. 2014; Decker et al. 2003; Neuner, Ambach, and Aichner 1999; Taschler and Neuner 2004).

Despite climate warming, unusual frost events may become more common (Lamichhane 2021). Warmer temperatures will cause deciduous plants to leaf out earlier putting them at risk for late spring frosts (Menzel, Helm, and Zang 2015; Zohner, Mo, and Renner 2018), and the slowdown of the Atlantic meridional overturning circulation may cause much cooler temperatures in Europe (Ditlevsen and Ditlevsen 2023; Jackson et al. 2015), posing new freezing risks to large sections of deciduous forest. Time‐lapse imagining and pixel brightness analysis is an affordable and simple method for observing freezing patterns and freezing initiation sites in leaves in situ, this method could be used to monitor natural freezing events in the field. Like the optical vulnerability method for determining embolism resistance from image subtraction, which has greatly increased the number of species for which we now have an ever‐growing data set of key water potentials of mortality (Cardoso et al. 2022), our method of pixel brightness analysis provides a simple means of widely sampling freezing tolerance thresholds across species, which could improve our modelling of forest and community responses to aseasonal freezing events and more accurately inform models of range changes into the future (Inouye 2000; Löffler 2007; Stuart et al. 2007; Tranquillini 1982; Walker et al. 2004). The high spatial resolution offered by this technique coupled with its ability to visualize freezing in situ during natural frost events, offers some advantages over traditional infrared camera methods that are often limited in field deplorability and high spatial resolution (Zalazar, Zypman, and Drori 2023). Our work also shows that when evaluating frost survival ex situ, it is critical to account for the rate of temperature decline and the rate of temperature increase upon thawing as rapid freezing and thawing can cause damage to leaves that may have survived if frozen slower.

## Supporting information

Supporting information.

Supporting information.

## Data Availability

The data that support the findings of this study are available from the corresponding author upon reasonable request.
