# Supplementary material for: Spatial and Temporal Freezing Dynamics of Leaves Revealed by Time‐Lapse Imaging
Source: Plant Cell Environ. 2024 Sep 10;48(1):164–75. doi: 10.1111/pce.15118 (PMC11615429; doi:10.1111/pce.15118)
Supplement: Supplementary file 1 — Supporting information. [file PCE-48-164-s002.docx]

Supplemental materials

Supplementary Video S1. Video depicting the raw time lapse of the 1 mm^2^ area of leaf through natural freezing and thawing on DOY 28, 2021. The figure below the movie shows the nocturnal course of leaf temperature (red) and relative pixel brightness of a 0.01 mm^2^ section of leaf containing mostly vein (black) or mesophyll (gray). The horizontal line marks 0°C. The insert depicts the area of the leaf from E in which the brightness was measured in with the black pixel being the section of leaf containing the vein and the gray section of leaf from the mesophyll.


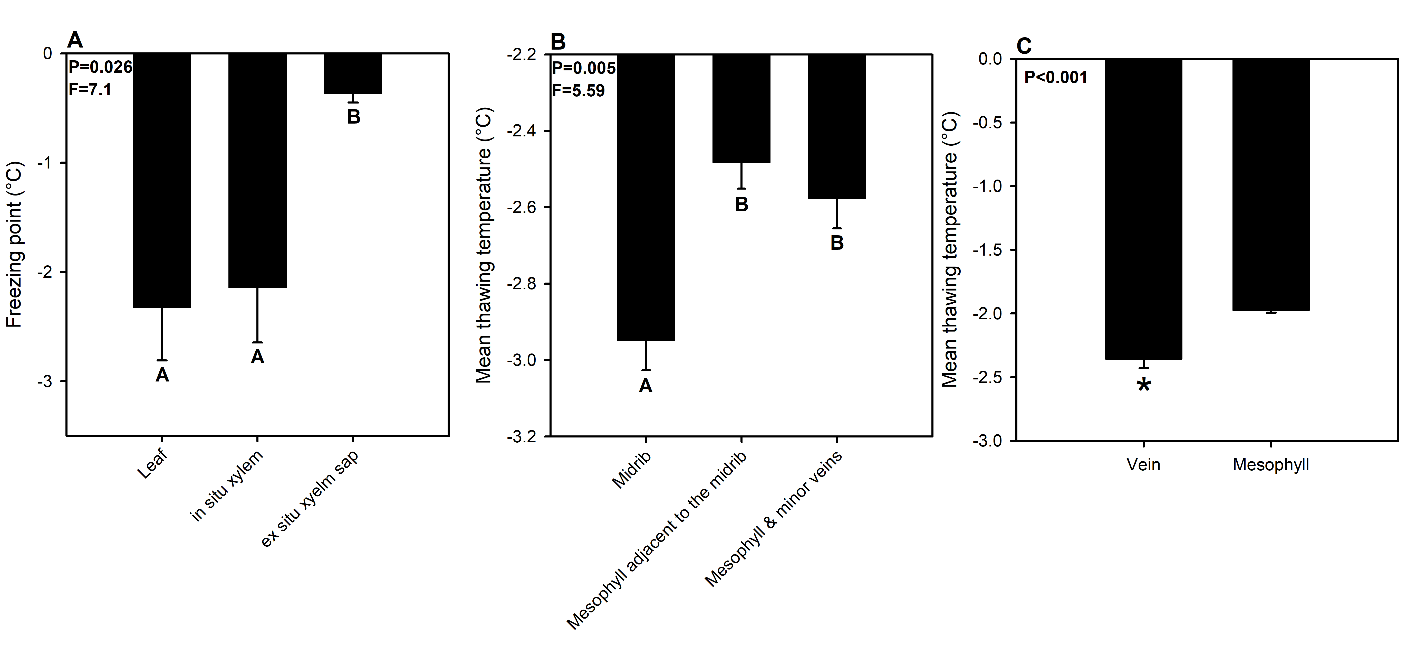


Supplementary Figure S1. (A) The mean (n=3) freezing temperature of *ex situ* leaves, exposed xylem tissue, and extracted xylem sap, the letters denote significant differences in means based on a one-way ANOVA and a Tukey's HSD post hoc test (p<0.05). (B) The difference in mean thawing temperatures between 0.124 mm^2^ sections of leaf that contain midrib, mesophyll adjacent to the midrib, and mesophyll and minor veins the letters denote significant differences in means based on a one-way ANOVA and a Tukey's HSD post hoc test (p<0.05). (C) The difference in mean thawing temperature between 0.01 mm^2^ sections of leaf that contain the veins and sections that contain the mesophyll. The ‘*’ indicates that they are significantly different based on a two-way student-T test.


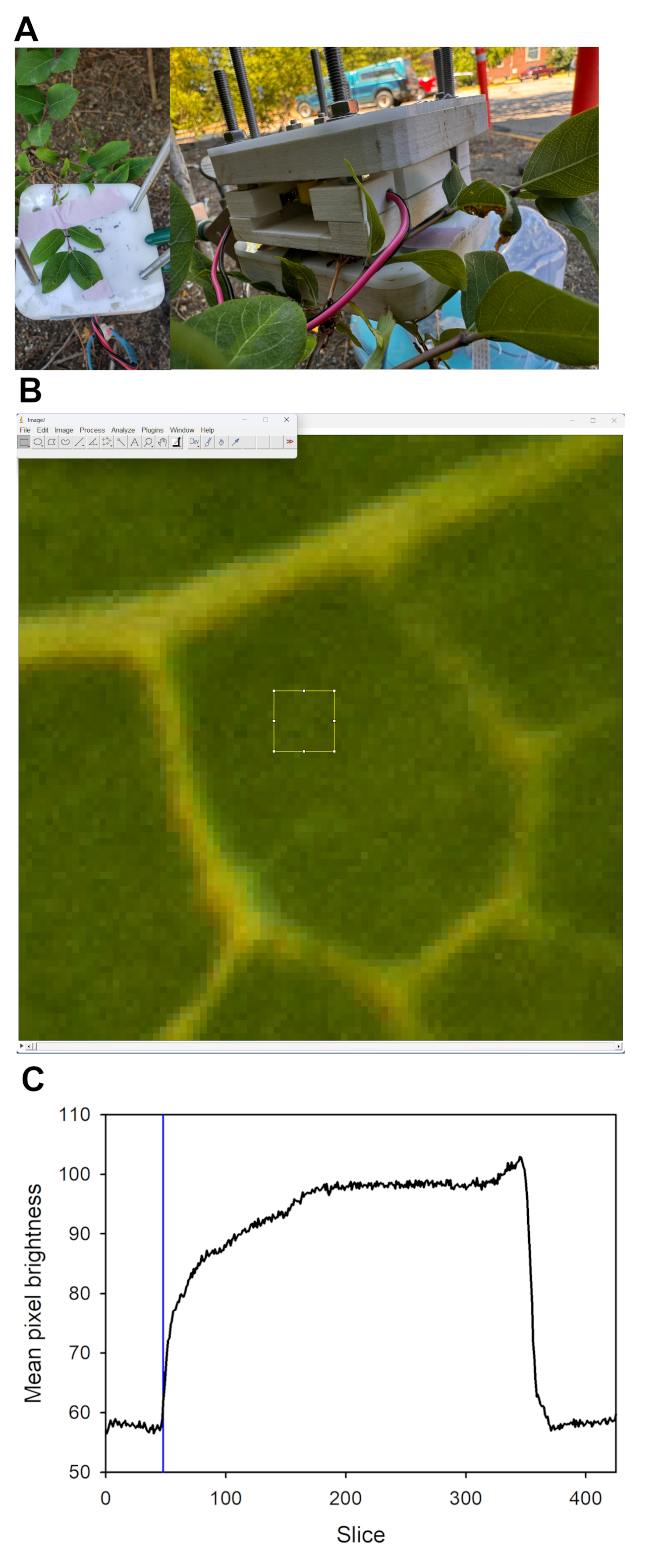


Supplementary Figure S2. (A) An example the field set up of the Raspberry Pi powered clamp on a leaf of *L. x purpusii,* with the more robust clamp open to the elements and the more sensitive Raspberry Pi kept in protective housing. (B) In ImageJ image analysis software the timelapse collected in the field is assembled into an image stack which is then broken up into smaller sections for more in depth spatial analysis, mean pixel brightness is then extracted. (C) Mean pixel brightness is plotted against image number in the stack and the freezing threshold is determined based an increase in mean pixel brightness of 10% (blue line).
